# Development of New Equation for Predicting State of Normometabolism from Cohort of Hospitalized Patients with Obesity

**DOI:** 10.3390/nu17030482

**Published:** 2025-01-29

**Authors:** Giuseppe Mazzola, Mariangela Rondanelli, Carlo Cattaneo, Alessandro Lazzarotti, Clara Gasparri, Gaetan Claude Barrile, Alessia Moroni, Francesca Mansueto, Leonardo Minonne, Simone Perna

**Affiliations:** 1 Endocrinology and Nutrition Unit, Azienda di Servizi alla Persona “Istituto Santa Margherita”, University of Pavia, 27100 Pavia, Italy; carlo.cattaneo03@universitadipavia.it (C.C.); alessandro.lazzarotti01@universitadipavia.it (A.L.); clara.gasparri01@universitadipavia.it (C.G.); gaetanclaude.barrile01@universitadipavia.it (G.C.B.); alessia.moroni02@universitadipavia.it (A.M.); francesca.mansueto01@universitadipavia.it (F.M.); leonardo.minonne01@universitadipavia.it (L.M.); 2 Department of Public Health, Experimental and Forensic Medicine, University of Pavia, 27100 Pavia, Italy; mariangela.rondanelli@unipv.it; 3 Department of Food, Environmental and Nutritional Sciences, Division of Human Nutrition, University of Milan, 20133 Milan, Italy

**Keywords:** obesity, normometabolic status, resting energy expenditure, predictive equation, diet therapy

## Abstract

**Background/Objectives:** Existing resting energy expenditure (REE) predictive equations, including Mifflin-St Jeor and Harris–Benedict, show limited accuracy, particularly in patients with a BMI over 35, often leading to overestimation or underestimation of REE. This study aimed to develop a new predictive equation specifically designed to identify normometabolic status in patients with obesity, enabling more precise qualitative assessments of basal metabolism through indirect calorimetry. **Methods:** A cohort of 89 hospitalized patients with obesity (BMI > 30) underwent REE measurement and comprehensive anthropometric assessments. Patients were classified as normometabolic if their REE was within ±10% of the Mifflin-St Jeor prediction or if their fat-free mass-specific REE fell between 23 and 30 kcal/kg. **Results:** The newly developed equation demonstrated high predictive accuracy (R^2^ = 0.923, root mean square error = 81.872 kcal/day), with a mean bias of −0.054 kcal/day and narrower limits of agreement (−156.834 to 156.725 kcal/day) compared to widely used models. **Conclusions:** These advancements could enhance follow-up and management of diet therapy in patients with obesity, allowing for a more tailored approach to their metabolic health over time.

## 1. Introduction

Obesity is a multifactorial condition resulting from a sustained positive energy balance, where caloric intake exceeds expenditure [1]. This condition is associated with an elevated risk of metabolic and cardiovascular disorders, type 2 diabetes, and certain cancers, representing a major challenge for global healthcare systems [2]. The accurate assessment of resting energy expenditure (REE), a critical component of energy balance, is essential for tailoring dietary interventions and optimizing clinical management in obese individuals.

Predictive equations, such as Mifflin-St Jeor and Harris–Benedict, are widely used to estimate REE but frequently lack precision, particularly in patients with a BMI exceeding 40 kg/m^2^. Errors in REE estimation can exceed 250–315 kcal/day in this population, rendering these equations less reliable in clinical practice [3,4]. Indirect calorimetry remains the gold standard for REE measurement, but its cost and logistical demands often limit its application in routine clinical settings [5]. These limitations are further exacerbated by the lack of a standardized definition for normometabolic status in obesity, which complicates metabolic evaluations and the interpretation of REE values.

The primary determinant of REE is fat-free mass (FFM), a metabolically active compartment comprising skeletal muscle and organs such as the liver and brain. Based on predictive equations and allometric models, the specific metabolic rate (KiK_iKi) of FFM is generally estimated at 23–24 kcal/kg/day [6,7]. However, under conditions of metabolic stress, such as sepsis, the KiK_iKi of FFM can rise significantly to 30–35 kcal/kg/day due to increased systemic inflammation and catabolic activity [8]. In contrast, fat mass (FM) exhibits a relatively constant metabolic rate of approximately 4–5 kcal/kg, irrespective of physiological or pathological states [6]. This disparity underscores the predominant role of FFM in driving interindividual variability in REE.

Despite these insights, significant challenges remain. Current predictive models and allometric equations fail to capture the complex interplay between body composition and metabolic rate, making it difficult to define normometabolism in obese individuals. Additionally, no consensus exists regarding the KiK_iKi value of FFM specific to obese populations. While data suggest that this value should range between 23 and 30 kcal/kg/day, variability across studies highlights the need for more precise and standardized methodologies to assess metabolic status in obesity [6,8].

To address the limitations of existing predictive equations, we developed a mathematical model based on a linear regression equation specifically designed to predict normometabolic status in patients with obesity. The primary objective of this study is to provide a novel tool for assessing whether a patient’s metabolic rate is normal, slowed, or accelerated relative to their body composition, a critical aspect currently overlooked by existing models. This model aims to complement indirect calorimetry by offering a practical and reliable method for evaluating metabolic health. By integrating this equation into clinical practice, we intend to support more precise dietary adaptations, enhance the phenotyping of obese individuals, and improve the long-term management of obesity through tailored interventions.

## 2. Materials and Methods

### 2.1. Study Design and Participant Selection

This cross-sectional observational study included 89 patients with obesity (BMI > 30 kg/m^2^), recruited from the inpatient units of the Santa Margherita Institute, Pavia, Italy, between January 2016 and January 2024.

Inclusion criteria required participants to be normometabolic obese individuals, defined by two primary conditions.

Eligible participants were classified as normometabolic based on the following inclusion criteria:

- Resting Energy Expenditure (REE) ≥ 90% and ≤110% of the value predicted by the Mifflin-St Jeor equation.
- Fat-free mass-specific REE (FFM Ki) between 23 and 30 kcal/kg, calculated using calorimetry and dual-energy X-ray absorptiometry (DXA).

Patients with conditions known to affect metabolism, such as uncontrolled thyroid disorders or recent weight-loss interventions, were excluded. 

### 2.2. Anthropometric, Body Composition Measurements, and Indirect Calorimetric Data

Anthropometric data included the following: body weight (kg), height (m), waist and hip circumferences (cm), and arm and calf circumferences (cm). Measurements were performed using calibrated electronic scales and stadiometers.

Body composition was assessed using the Lunar Prodigy DXA system with Prodigy software (GE Healthcare encore V17), providing precise estimates of fat mass (FM), fat-free mass (FFM), and visceral adipose tissue (VAT). Adherence to standardized protocols ensured accuracy and repeatability.

REE was measured using the COSMED Q-NRG calorimeter in canopy mode. Testing was conducted in the morning after a 12 h fasting period and a 30 min resting phase in a thermoneutral environment. Measurement criteria included the following:

- A minimum of 5 min of steady-state data,
- Coefficients of variation for VO_2_ and VCO_2_ < 4%,
- Maintenance of steady-state conditions for at least 3 consecutive minutes.

REE calculations employed the Weir equation, using oxygen consumption (VO_2_) and carbon dioxide production (VCO_2_) values [5].

### 2.3. Statistical Analysis

All statistical analyses were performed using JASP software (0.19.3.0 version) Descriptive statistics summarized baseline characteristics, providing means and standard deviations for continuous variables. The following analytical approaches were employed:

#### 2.3.1. Correlation and Residual Analysis

Correlations between anthropometric variables and metabolic parameters were analyzed using Pearson’s correlation coefficient to evaluate the strength and direction of linear relationships among continuous variables. Specifically, correlations were examined between weight, height, age, gender, and body circumferences (waist, hip, arm, and calf) and REE measured via indirect calorimetry. Significant correlations (*p* < 0.05) were further explored to assess their relevance in predicting REE. Residuals from the regression model were examined for normality using the Shapiro–Wilk test and graphically assessed via histograms and Q-Q plots. Homoscedasticity was verified by plotting residuals against predicted values to ensure uniform variance.

#### 2.3.2. Regression Analysis

To construct a predictive model for REE, multiple linear regression analysis was employed. Anthropometric variables such as weight, height, age, gender, and body circumferences were used as independent variables. The model was developed using a stepwise procedure, which progressively included the most statistically significant variables to enhance predictive capability. Variable selection was based on minimizing the standard error and maximizing the coefficient of determination (R^2^).

#### 2.3.3. Bland–Altman Analysis

This method assessed agreement between measured REE (via indirect calorimetry) and predicted values from the established equations. Agreement between measured and predicted REE was assessed for the developed equation and the following predictive equation: Mifflin-St Jeor, Harris–Benedict, Bernstein, Henry, Ravussin, Cunningham, Owen. Mean bias (mean difference) and 95% limits of agreement provided insights into the systematic error and variability of each predictive equation.

#### 2.3.4. Paired *t*-Tests

Paired sample *t*-tests compared the mean differences (bias) between the REE predicted by each equation and the measured REE. The *t*-test results provided insights into systematic overestimation or underestimation by each model. Differences were considered statistically significant at *p* < 0.05.

### 2.4. Ethical Considerations

This study was conducted in accordance with the Declaration of Helsinki and approved by the Ethics Committee of the University of Pavia (Approval Code: 6723/22052019). All participants provided written informed consent prior to enrollment.

## 3. Results

### 3.1. Baseline Characteristics of the Sample

The selected sample included 89 normometabolic patients with obesity, of which 69.6% were female (n = 62) and 30.4% were male (n = 27). All patients presented with a BMI > 30, confirming their classification as obese based on the criteria set by the World Health Organization (WHO, 2020). The patients’ anthropometric measurements and body composition data are summarized in Table 1.

### 3.2. Normality Analysis and Pearson Correlation

**Figure 1:**
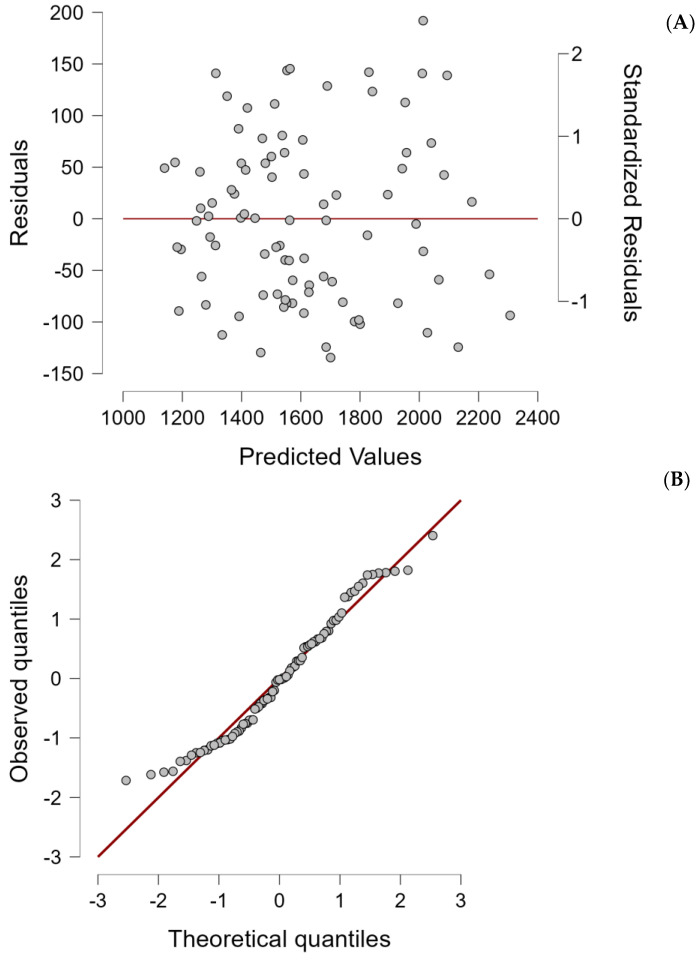
(**A**) Graphical analysis of residuals vs. predicted values and (**B**) Q-Q plot of standardized residuals.

The normality of the sample was assessed using the Shapiro–Wilk test (Table 2), a graphical analysis of residuals versus predicted values (Figure 1A), and a Q-Q plot of standardized residuals (Figure 1B).

The Shapiro–Wilk test results (Table 2) revealed significant deviations from normality for multiple variables, with RMR Calorimetry T0 showing a statistic of 0.948 (*p* = 0.001) and FFM T0 presenting a more pronounced departure with a statistic of 0.937 (*p* < 0.001). Other variables, such as Height and Waist Circumference, showed borderline significance (*p* = 0.022 and *p* = 0.901, respectively), suggesting varying degrees of normality across the dataset. These results indicate that the assumption of normality is not fully met for key predictors and outcome variables.

The Q-Q plot of standardized residuals (Figure 1B) provides additional visual confirmation, where slight deviations from the expected diagonal line are observed, particularly in the tails. This indicates the presence of outliers or a non-normal distribution of residuals. The residuals vs. predicted values plot (Figure 1A) further highlights potential issues, displaying a non-random pattern of residuals, which may reflect heteroscedasticity or systematic bias in the model predictions.

These deviations from normality likely stem from specific characteristics of the sample. The relatively small sample size may limit the robustness of the statistical analyses, while the uneven demographic distribution introduces additional challenges. In particular, the cohort contains a disproportionate number of older individuals, predominantly female participants, and individuals with higher BMI values (notably in the 35–40 kg/m^2^ and 40–45 kg/m^2^ ranges), compared to those in the 30–35 kg/m^2^ range. These factors contribute to the skewed distribution of variables and residuals, affecting the normality assumptions of the regression model.

Despite this non-normality in certain critical variables, the regression model remains theoretically robust. The graphical analysis of residuals further supports the adequacy of the model by demonstrating a random distribution of residuals without systematic patterns. However, the lack of normality in these key variables has likely reduced the predictive accuracy of the model, underscoring the importance of larger and more diverse samples in future validations to enhance reliability and generalizability.

The analysis of Pearson’s correlations revealed a strong positive relationship between REE and FFM (r = 0.928; *p* < 0.001), confirming that FFM is the primary determinant of REE due to its high metabolic activity. REE also showed a moderate correlation with FM (r = 0.443; *p* < 0.001), indicating a smaller but measurable contribution of fat tissue to energy expenditure. A similar moderate positive correlation was observed between REE and waist circumference (r = 0.662; *p* < 0.001), highlighting the metabolic impact of visceral fat. Conversely, a moderate negative correlation was identified between REE and age (r = −0.404; *p* < 0.001), reflecting the decline in energy expenditure with aging, likely due to sarcopenia and reduced metabolic activity. Lastly, sex was significantly correlated with REE (r = 0.707; *p* < 0.001), with men exhibiting higher REE values, attributed to their greater FFM and hormonal differences. These findings underline the multifactorial determinants of REE in obese individuals. Full correlation data are detailed in Table 3.

### 3.3. Regression Model Development

A stepwise linear regression identified weight, sex, height, and age as significant predictors of REE, as shown in Table 4. The final regression model (M_4_) identified weight, sex, height, and age as the most significant predictors of resting energy expenditure (REE) in normometabolic patients with obesity. This model demonstrated a high explanatory capacity, with an R^2^ of 0.923, indicating that 92.3% of the variability in REE could be accounted for by these variables. Among the predictors, weight emerged as the most influential, while the inclusion of age further enhanced the model’s accuracy, reflecting the metabolic impact of aging. The root mean square error (RMSE) of 81.872 kcal/day underscores the precision of this model in predicting REE.

**Equation elaborated from Model (M_4_)**

*RMR predicted* (kcal/day) = −* + (*×*Weight*(Kg)) + (*×1 *for men or* ×0 *for women*) + (*×*Height* (m)) − (*×*Age* (years))

### 3.4. Bland–Altman Analysis and Paired t-Tests

**Figure 2:**
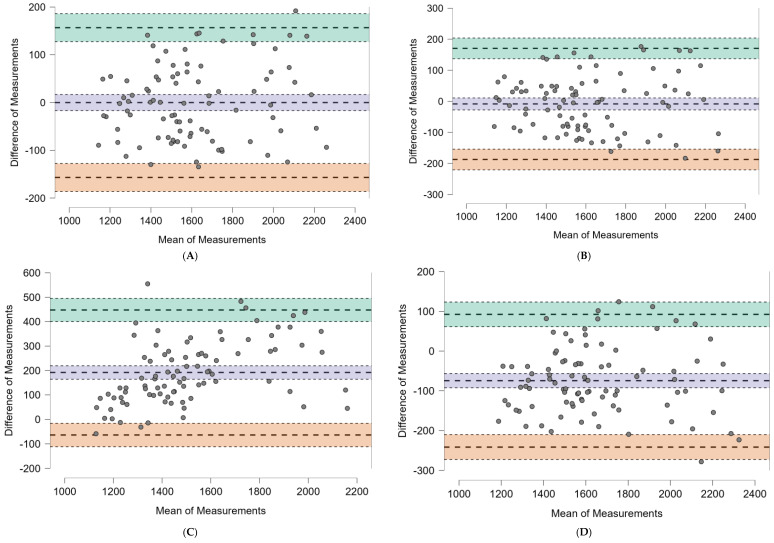

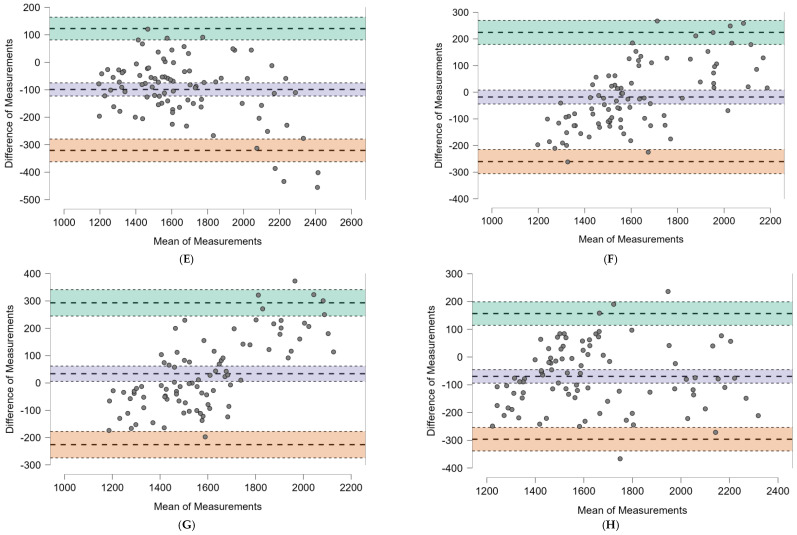
Bland–Altman plots of the different predictive equations for REE analyzed and our equation: (**A**) Our equation, (**B**) Mifflin-St Jeor, (**C**) Bernstein, (**D**) Henry, (**E**) Harris–Benedict, (**F**) Cunningham, (**G**) Ravussin, and (**H**) Owen.

**Bland–Altman Analysis:** The predictive accuracy of the newly developed equation was compared against existing models for estimating resting energy expenditure (REE) using Bland–Altman analysis (Figure 2 and Table 5). The new equation demonstrated a mean bias of −0.054 kcal/day with relatively narrow limits of agreement (−156.834 to 156.725 kcal/day), indicating consistent performance across the sample. In contrast, other commonly used equations, such as Mifflin-St Jeor, Harris–Benedict, and Bernstein, exhibited wider limits of agreement (LOA) and significant biases. For instance, the Mifflin-St Jeor equation displayed a mean bias of −8.452 kcal/day, but with a broader LOA (−187.390 to 170.486 kcal/day). The Bernstein equation showed notable overestimation, with a mean bias of +191.846 kcal/day and an extremely wide LOA (−64.022 to 447.714 kcal/day).

The Harris–Benedict equation demonstrated a systematic underestimation, with a mean bias of −98.838 kcal/day and LOA ranging from −320.722 to 123.046 kcal/day. Other equations, such as those by Henry and Owen, also displayed systematic underestimations, while the equations by Ravussin and Cunningham performed moderately better. These latter models showed biases of +33.705 kcal/day and −17.942 kcal/day, respectively, though both exhibited greater variability compared to the newly developed model.

The Bland–Altman plots (Figure 2) visually depict these findings, highlighting the tighter agreement and reduced variability of the new equation compared to other models.

**Paired *t*-tests**: Paired *t*-tests were conducted to evaluate differences in bias between the new equation and established predictive equations (Table 6). The analysis revealed that the new equation outperforms others in terms of precision and consistency, with the smallest mean bias (−0.054 kcal/day) and the narrowest limits of agreement (−156.834 to 156.725 kcal/day). These findings underscore the superior accuracy of the new equation compared to the widely used Mifflin-St Jeor equation, which exhibited a slightly higher bias (−8.452 kcal/day) and broader limits of agreement (−187.390 to 170.486 kcal/day). The bias difference between the new equation and Mifflin-St Jeor was not statistically significant (*p* = 0.075), further highlighting the robustness of the new equation. Its narrower limits of agreement indicate greater precision in predicting REE across the cohort, making it a more reliable tool in clinical applications where reducing variability is crucial, such as metabolic monitoring during weight-loss interventions.

In contrast, other predictive equations showed significant limitations. The Bernstein equation presented a substantial overestimation bias (+191.846 kcal/day; *p* < 0.001) with the broadest limits of agreement, while the Harris–Benedict equation significantly underestimated REE (−98.838 kcal/day; *p* < 0.001). Similarly, the Henry and Owen equations demonstrated notable underestimations, with biases of −74.547 kcal/day and −69.786 kcal/day, respectively (*p* < 0.001 for both). Although the Cunningham equation exhibited a relatively smaller bias (−17.942 kcal/day), its wider variability limits its precision in clinical settings.

Overall, the new equation stands out as the most precise and consistent model, with the lowest mean bias and the narrowest limits of agreement among all equations analyzed, reaffirming its superiority as a predictive tool for REE estimation in patients with obesity.

## 4. Discussion

### 4.1. Accuracy and Clinical Relevance of the New Equation

The newly developed equation exhibited robust predictive accuracy for estimating REE in normometabolic obese individuals, as demonstrated by its low mean bias and narrow limits of agreement when compared to REE measured via indirect calorimetry. These findings suggest that the model performs with reduced variability relative to existing predictive equations, which are commonly used in clinical practice.

The comparative analysis revealed that the new equation outperformed traditional predictive models in terms of accuracy and risk of bias within the sample. For instance, while the Mifflin-St Jeor equation performed relatively well, its broader limits of agreement and increased variability indicate less precision compared to the new model. Additionally, the Mifflin-St Jeor equation exhibited a slightly lower mean bias than the new model; however, this difference was not statistically significant, likely due to the limited sample size. Other predictive equations, such as Bernstein, Harris–Benedict, and Owen, demonstrated higher mean biases and wider limits of agreement, further highlighting the superior performance of the new equation in this cohort.

Overall, the new equation demonstrated the best alignment with measured REE values in the sample, providing both improved accuracy and reduced bias compared to existing models. This characteristic underscores its potential utility as a reliable tool in clinical practice, particularly in scenarios where direct calorimetry is not feasible.

### 4.2. Practical Implications and Applications

The primary advantage of the newly developed equation lies in its ability to evaluate the normometabolic status of patients with obesity. Unlike traditional predictive models, which focus solely on estimating REE, this equation offers a framework for determining whether a patient’s metabolism is normal, slowed, or accelerated relative to their body composition. This unique capability provides a critical tool for phenotyping obese individuals and adds significant value to the metabolic assessment of this population. By assessing normometabolic status, the equation facilitates a more nuanced understanding of metabolic variability among patients with obesity. This is particularly relevant for identifying subclinical chronic inflammation or the impact of comorbidities, such as diabetes, on metabolic function. Such insights are crucial for tailoring interventions and understanding the broader clinical implications of metabolic health in obesity.

The ability to evaluate normometabolic status has important practical implications in both clinical and research contexts. Clinically, this evaluation can support the design of personalized dietary interventions. For instance, if a patient’s metabolism is classified as slowed relative to their normometabolic status, adjustments to caloric intake or macronutrient composition—such as increasing dietary protein—could be considered to mitigate potential muscle loss and support metabolic health. Moreover, monitoring changes in normometabolic status during long-term interventions can provide valuable feedback on the effectiveness of dietary strategies. For example, a decline in normometabolic status during a very-low-calorie ketogenic diet (VLCKD) might indicate the need for temporary caloric increases (reverse dieting) to prevent metabolic adaptation and optimize outcomes [9].

From a research perspective, the equation opens new avenues for investigating how specific comorbidities influence the metabolic quality of patients with obesity. This is a relatively unexplored area that could provide critical insights into the interplay between obesity, inflammation, and systemic metabolic dysregulation. By enabling a standardized assessment of normometabolic status, the equation could facilitate studies aimed at understanding the qualitative effects of chronic conditions on energy expenditure.

### 4.3. Limitations of the Proposed Equation and Future Direction

Despite its promising performance, the new equation has several limitations. First, the relatively small sample size may restrict the generalizability of the findings, highlighting the need for validation in larger and more diverse populations to confirm their broader applicability. A second limitation lies in the demographic composition of the sample, which included a predominance of female participants (66.7%). This imbalance might affect the equation’s performance in predominantly male populations, as metabolic differences between genders, although partially accounted for by including sex as a variable, may not be fully addressed. The age distribution of the sample, with a mean age of 61 years, raises additional concerns. Younger individuals, who often exhibit different metabolic rates and body compositions, were underrepresented. This limits the equation’s utility in populations with a wider age range. Moreover, some variables did not follow normal distributions, as evidenced by the Shapiro–Wilk test. Although residual analysis suggested that the regression model was appropriate, deviations from normality could affect the reliability of predictions, particularly at the extremes of the dataset.

To address these limitations, further studies are essential, involving larger and more diverse cohorts that better reflect the variability in demographic and clinical profiles. Such research would not only refine the equation but also validate its utility across broader populations. Future efforts should prioritize expanding cohort diversity by including younger individuals, patients with significant metabolic disorders, and a more balanced representation of genders. This would improve the generalizability of the equation and ensure its applicability to a wider range of clinical scenarios. Additionally, longitudinal studies are needed to evaluate the equation’s performance over time, particularly in dynamic contexts such as weight-loss programs or metabolic interventions. This approach would establish its reliability and effectiveness in monitoring metabolic changes and guiding long-term therapeutic strategies.

These steps would not only solidify the equation’s role in clinical practice but also expand its relevance in cutting-edge metabolic research.

## 5. Conclusions

The newly developed equation for predicting REE in normometabolic patients with obesity demonstrated superior accuracy and reliability compared to traditional models, including Mifflin-St Jeor and Bernstein. With reduced bias and narrower limits of agreement, the new equation emerged as the most precise tool for estimating REE in this specific population. These characteristics make it particularly valuable for clinical scenarios where indirect calorimetry is unavailable, supporting personalized dietary and therapeutic strategies essential for effective obesity management.

However, the equation has some limitations that require consideration. The relatively small sample size (n = 89) may limit the generalizability of the findings, necessitating validation in larger and more diverse cohorts. Additionally, the demographic imbalance, with a predominance of older and female participants, restricts its applicability to younger or predominantly male populations, where metabolic rates and body composition differ significantly. Furthermore, deviations from normality in some variables might reduce predictive reliability, particularly at dataset extremes.

Future research should focus on several key areas to strengthen the applicability and impact of the proposed equation. Firstly, studies involving larger and more demographically diverse cohorts are essential to confirm the generalizability of the findings. Specifically, including younger individuals, males, and patients with varied metabolic conditions will provide a more comprehensive understanding of the equation’s robustness across different populations.

Secondly, longitudinal investigations are needed to assess how the equation performs over time, particularly in dynamic clinical contexts such as weight-loss programs, metabolic rehabilitation, and the management of chronic conditions like type 2 diabetes. Such studies will clarify the utility of the equation in monitoring metabolic adaptations and guiding long-term therapeutic strategies.

Finally, integrating the equation into interventional studies could provide valuable insights into its practical utility in personalizing dietary interventions and improving clinical outcomes. For instance, evaluating how the equation informs caloric and macronutrient adjustments during very low-calorie diets or ketogenic interventions could further demonstrate its relevance in routine clinical practice. These efforts will refine the equation’s utility and ensure its applicability across a broader spectrum of clinical scenarios, ultimately advancing metabolic assessment and personalized care for patients with obesity.

## Figures and Tables

**Table 1:** General characteristics of the sample.

Characteristic	Mean ± SD	Median (Range)
**Total sample**		
Age (years)	61.7 ± 11.8	63.0 (24.0–80.0)
Weight (kg)	104.3 ± 17.9	103.6 (69.8–151.5)
Height (m)	1.60 ± 0.10	1.58 (1.41–1.80)
BMI (kg/m^2^)	40.85 ± 6.48	40.30 (29.61–58.40)
Waist circumference (cm)	123.9 ± 13.1	123.0 (94.0–160.0)
Hip circumference (cm)	125.7 ± 12.9	125.5 (101.5–155.0)
FFM (kg)	51.2 ± 9.6	48.7 (35.9–76.3)
FM (kg)	49.2 ± 11.3	48.7 (28.1–72.4)
**Male**		
Age (years)	61.4 ± 14.6	62.0 (22.0–83.0)
Weight (kg)	114.1 ± 18.8	117.7 (81.2–151.5)
Height (m)	1.69 ± 0.07	1.70 (1.53–1.80)
BMI (kg/m^2^)	39.7 ± 5.5	40.5 (29.6–50.5)
Waist circumference (cm)	129.9 ± 12.6	131.0 (105.0–160.0)
Hip circumference (cm)	119.2 ± 11.5	119.0 (101.5–141.0)
FFM (kg)	62.0 ± 7.8	63.2 (47.7–76.3)
FM (kg)	47.2 ± 12.6	48.7 (28.1–72.4)
**Female**		
Age (years)	64.1 ± 10.9	66.0 (31.0–80.0)
Weight (kg)	99.9 ± 15.8	97.0 (69.8–144.0)
Height (m)	1.56 ± 0.08	1.56 (1.41–1.79)
BMI (kg/m^2^)	41.4 ± 6.9	40.1 (31.1–58.4)
Waist circumference (cm)	121.1 ± 12.5	120.3 (94.0–150.0)
Hip circumference (cm)	128.5 ± 12.6	128.0 (108.5–155.0)
FFM (kg)	46.3 ± 5.3	46.3 (35.9–60.9)
FM (kg)	50.1 ± 10.7	47.2 (32.5–72.2)

**Table 2:** Shapiro–Wilk Test Results.

Variable	Shapiro–Wilk Statistic	*p*-Value
RMR Calorimetry (kcal/day)	0.948	0.001
Height (m)	0.967	0.022
Weight Kg	0.981	0.224
Arm cm	0.966	0.029
Calf cm	0.953	0.005
Waist cm	0.992	0.901
Hips (cm)	0.977	0.214
FFM (g)	0.937	<0.001
FM (g)	0.972	0.054
VAT (g)	0.939	0.002
BMI	0.967	0.023

**Table 3:** Pearson correlation analysis of variables collected in patients with obesity.

Variable	RMR Calorimetry T0 (kcal/day)	FM T0 (g)	FFM T0 (g)	Entry Hips	Waist T0	Age (Years)	Gender
**1. REE (kcal/day)**	**Pearson’s r**	—	0.441 ***	0.924 ***	0.081	0.661 ***	−0.375 ***
	*p*-value	—	<0.001	<0.001	0.506	<0.001	<0.001
	**Spearman’s rho**	—	0.466 ***	0.898 ***	0.137	0.659 ***	−0.368 ***
	*p*-value	—	<0.001	<0.001	0.258	<0.001	<0.001
**2. FM (g)**	**Pearson’s r**	0.441 ***	—	0.238 *	0.820 ***	0.734 ***	−0.105
	*p*-value	<0.001	—	0.024	<0.001	<0.001	0.329
	**Spearman’s rho**	0.466 ***	—	0.306 **	0.808 ***	0.748 ***	−0.146
	*p*-value	<0.001	—	0.004	<0.001	<0.001	0.172
**3. FFM (g)**	**Pearson’s r**	0.924 ***	0.238 *	—	−0.047	0.550 ***	−0.388 ***
	*p*-value	<0.001	0.024	—	0.697	<0.001	<0.001
	**Spearman’s rho**	0.898 ***	0.306 **	—	−7.352 × 10^−4^	0.585 ***	−0.369 ***
	*p*-value	<0.001	0.004	—	0.995	<0.001	<0.001
**4. Hip (cm)**	**Pearson’s r**	0.081	0.820 ***	−0.047	—	0.632 ***	0.101
	*p*-value	0.506	<0.001	0.697	—	<0.001	0.404
	**Spearman’s rho**	0.137	0.808 ***	−7.352 × 10^−4^	—	0.607 ***	0.048
	*p*-value	0.258	<0.001	0.995	—	<0.001	0.673
**5. Waist (cm)**	**Pearson’s r**	0.661 ***	0.734 ***	0.550 ***	0.632 ***	—	−0.023
	*p*-value	<0.001	<0.001	<0.001	<0.001	—	0.826
	**Spearman’s rho**	0.659 ***	0.748 ***	0.585 ***	0.607 ***	—	−0.038
	*p*-value	<0.001	<0.001	<0.001	<0.001	—	0.712
**6. Age (years)**	**Pearson’s r**	−0.375 ***	−0.105	−0.388 ***	0.101	−0.023	—
	*p*-value	<0.001	0.329	<0.001	0.404	0.826	—
	**Spearman’s rho**	−0.368 ***	−0.146	−0.369 ***	0.082	−0.038	—
	*p*-value	<0.001	0.172	<0.001	0.501	0.712	—
**7. Gender**	**Pearson’s r**	0.704 ***	−0.120	0.766 ***	−0.332 **	0.314 **	−0.105
	*p*-value	<0.001	0.265	<0.001	0.005	0.003	0.327
	**Spearman’s rho**	0.663 ***	−0.110	0.720 ***	−0.325 **	0.300 **	−0.074
	*p*-value	<0.001	0.304	<0.001	0.006	0.005	0.488

REE: resting energy expenditure (kcal/day); FM: fat mass (g); FFM: fat-free mass (g). Correlation significance levels: * *p* < 0.05, ** *p* < 0.01, *** *p* < 0.001.

**Table 4:** Linear Regression Model Performance.

Model	Predictors Included	R^2^	Adjusted R^2^	RMSE	Sum of Squares	*df*	Mean Square	F	*p*
**M_1_**	Weight (Kg)	0.704	0.701	157.922	5.168 × 10⁶	1	5.168 × 10⁶	207.226	<0.001
**M_2_**	Weight (Kg), Gender	0.884	0.882	99.279	6.490 × 10⁶	2	3.245 × 10⁶	329.243	<0.001
**M_3_**	Weight (Kg), Gender, Height (m)	0.918	0.915	84.218	6.735 × 10⁶	3	2.245 × 10⁶	316.519	<0.001
**M_4_**	Weight (Kg), Gender, Height (m), Age (years)	0.923	0.920	81.872	6.775 × 10⁶	4	1.694 × 10⁶	252.678	<0.001
**Coefficients for Model (M_4_).**									
**Predictor**			**t**						***p***
(Intercept)			−1.332						0.187
Weight (Kg)			16.292						<0.001
Gender			8.651						<0.001
Height (m)			4.780						<0.001
Age (years)			−2.438						0.017

R^2^: coefficient of determination, Adjusted R^2^: adjusted coefficient of determination, RMSE: root mean square error, Predictors Included: variables included in the model, Sum of Squares: total variance explained by the model, *df*: degrees of freedom, Mean Square: average variance per predictor, F: F-statistic, *p*: *p*-value for significance, t: t-statistic, *p*: *p*-value for hypothesis testing.

**Table 5:** Bland–Altman Analysis of Predictive Equations.

Equation	Mean Bias (kcal/day)	95% Limits of Agreement (kcal/day)
Equation from M4 model	−0.054	−156.834 to 156.725
Mifflin-St Jeor	−8.452	−187.390 to 170.486
Bernstein	+191.846	−64.022 to 447.714
Harris–Benedict	−98.838	−320.722 to 123.046
Henry	−74.547	−241.460 to 92.366
Ravussin	+33.705	−225.842 to 293.253
Cunningham	−17.942	−260.408 to 224.523
Owen	−69.786	−296.256 to 156.684

**Table 6:** Paired *t*-tests Comparing Predictive Models.

Equation	*p*-Value (Bias vs. Measured REE)
Mifflin-St Jeor	0.414
Bernstein	<0.001
Harris–Benedict	<0.001
Henry	<0.001
Ravussin	0.008
Cunningham	0.161
Owen	<0.001

## Data Availability

The data presented in this study are available on request from the corresponding author. The data are not publicly available due to privacy restrictions.
